# Assessment of sleep and stress level in individuals with chronic pain

**DOI:** 10.5935/1984-0063.20220043

**Published:** 2022

**Authors:** Marcela Cavalcante, Carla Daltro, Durval Kraychete, Martha Cavalcante Castro

**Affiliations:** 1 Professor at EBMSP, Salvador, Bahia, Brazil.; 2 Professor at the Federal University of Bahia, Salvador, Bahia, Brazil.; 3 Professor at the Federal University of Bahia, Salvador, Bahia, Brazil.; 4 Associate Professor I at the Federal University of Bahia and EBMSP, Salvador, Bahia, Brazil.

**Keywords:** Chronic Pain, Stress, Physiological, Sleep, Anxiety, Depression

## Abstract

**Introduction:**

The experience of living with chronic pain allows for the appearance of changes in sleep patterns, mood, and stress levels.

**Objective:**

To describe the phases of stress and the quality of sleep in patients with chronic pain.

**Material and Methods:**

Cross-sectional study carried out at the pain clinic of the HUPES Complex, Salvador-Bahia. Data collection between March 2016 and November 2017. Instruments: Sociodemographic questionnaire, Numerical Pain Scale (EVN), Mini-Sleep Questionnaire (MSQ), and Stress Symptoms Inventory for LIPP adults (ISSL). Categorical variables were expressed by absolute and relative frequency and quantitative variables by means and standard deviation (SD). The comparison of categorical variables was performed using the chi-square test. Values of *p*<0.05 were considered statistically significant.

**Results:**

Mean age (standard deviation) of 50.0 (10) years, 89.6% of whom were female. Predominance of people with a partner, with religion, high school, and unemployed or removed by the INSS. They have severe sleep disorders, severe pain, and the presence of stress in the resistance phase. Most subjects reveal that they have improved with the treatment and have moderate self-esteem and personal satisfaction, despite the presence of anxious and depressive symptoms.

**Conclusion:**

Chronic pain has a very significant impact on life, increasing the level of stress, compromising and limiting daily activities, and showing more presence of anxious and depressive symptoms in people who suffer from chronic pain.

## INTRODUCTION

Chronic pain is a major public health problem, as it generates considerable impact that directly affects the health and economic systems^1^. It is estimated that, in Brazil, there are thirty to forty percent of people affected by chronic pain. Of these, pain is the biggest cause of absenteeism, sick leave, sick pay, labor compensation, and low productivity at work^2,3^.

The chronicity of pain is related to significant losses in the areas of sexuality, interpersonal relationships, working life, social interactions, self-care, in addition to the worsening in the quality of sleep patterns and stress^2^.

Pain is a complex stressor that presents significant challenges for society. It is necessary to identify the damage that pain causes in its entirety and to offer adequate treatment for individuals’ proper rehabilitation^4^.

Stress causes physiological and psychological changes. The physiological components are those various symptoms of body arousal such as: increased heart rate, increased blood pressure, wheezing, and dry mouth. The psychological components involve thoughts, emotions, and behaviors. These components are determined by the cognitive assessments that are made of the perceived threat^5,6^.

When the experience of stress is acute, that is, fast and transient the body can only resume homeostasis when the situation is under control. In the case of chronic stress, the individual’s vulnerability in acquiring diseases is increased^7^. According to a series of biological and environmental factors, each individual will respond to stress in different ways with greater or lesser repercussions to the organism^7,8^.

The stress system can also be activated after an injury. Painful perception is transmitted through specific nociceptive pathways to the cerebral cortex, making connections at various locations in the central nervous system, including the hypothalamus and the hypothalamic-pituitary-adrenal axis, which is related to the fight or flight response used in overcoming threats to an organism^9,10^.

Although stressful events can be an inevitable part of life, being exposed to long-term pain can intensify sympathetic and neuroendocrine activity. Stress and pain are related and these processes are probably of multidirectional and cyclical natures.

People with chronic pain have a high level of stress psychological, which consequently can compromise the quality of sleep, increasing the intensity and making pain management more difficult^11^. Thus, the objective of this work is to describe the phases of stress and the quality of sleep in patients with chronic pain and investigate the relationship between these variables.

## MATERIAL AND METHODS

### Study

Observational study with descriptive cross-sectional design with a consecutive sample. The patients were treated at the Pain Clinic - C-HUPES (*Complexo Hospitalar Professor Edgard Santos*) in Salvador-Bahia from June 2016 To March 2019. Patients of both genders and between the ages of 18 and 80 years were included in the study. We excluded subjects who had difficulty understanding the study, i.e., individuals who did not complete the protocol and patients with cancer-type pain. The project was approved by the Research Ethics Committee of HUPES (Professor Edgard Santos University Hospital), number: 1.496.509.

All new patients who entered the service were invited to participate in the research. They performed the interview and application of the scales in a single moment by the same researcher.

### Instruments

A questionnaire of sociodemographic and clinical data was administered to participants. The questionnaire provided data on the participants that included age, gender, marital status, education, religion, profession, socioeconomic status, level of personal satisfaction, family data, spirituality/religion, as well as data related to pain, i.e., diagnosis, intensity, time of greatest pain onset, and treatments instituted in the course of illness. The visual numerical scale (EVN) was used to assess pain, consisting of a line, graded from 0 to 10, where zero represented absence of pain and ten the worst pain imaginable. The values obtained were categorized as follows: 0 = no pain; 1, 2 and 3 = mild pain; 4, 5 and 6 = moderate pain; 7, 8 and 9 = severe or severe pain; and 10 = unbearable pain.

The mini-sleep questionnaire (MSQ) was used to assess sleep. The instrument consisted of ten questions, of which six items assessed symptoms of excessive daytime sleepiness and the other four expressed sleep quality^12^. Each item received a score ranging from 1 to 7: (1) never; (2) very rarely; (3) rarely (4) sometimes; (5) frequently; (6) very often; (7) always. The sum of the ten responses generated a numerical score that was classified into four categories for the degree of sleep disorders^13^. The items that were assessed by the MSQ are symptoms of excessive daytime sleepiness, falling asleep during the day, nonrestorative sleep, presence of morning fatigue, difficulty falling asleep, snoring, waking up too early, waking up at night, using sleeping medication, having headache head on awakening, and unexplained chronic fatigue. The MSQ categorization was 10 to 24 points (good sleep), 25 to 27 points (mild disorder), 28 to 30 points (moderate disorder), and above 30 points (severe disorder).

The inventory of symptoms of stress for adults (ISSL) consisted of three tables that refer to the phases of stress that are alert, resistance, almost exhaustion, and exhaustion. The first table referring to the alert phase consisted of 15 items and referred to the physical or psychological symptoms that the person had experienced in the last 24 hours. The second was composed of ten physical and five psychological symptoms (related to the symptoms experienced in the last week) and referred to the phases of resistance and/or near exhaustion. The third table referred to the exhaustion phase and consisted of 12 physical and 11 psychological symptoms (experienced in the last month). In total, ISSL presented 37 items that were a somatic in nature and 19 items that were psychological in nature^14^.

The mini-sleep questionnaire (MSQ) scales was used to assess sleep; the visual numerical scale (EVN) was used to assess pain and the inventory of symptoms of stress for adults (ISSL) were validated for use in the Brazilian context.

### Data analysis

Data analysis was performed using the SPSS Statistical Program, version 17.0. Categorical variables were expressed by absolute and relative frequency and quantitative variables by means and standard deviation (SD). The comparison of categorical variables was performed using the chi-square test or Fisher’s exact test. To study the correlation between ordinal variables, Spearman’s correlation test was used. The tests were two-tailed and only applied if the assumptions were met. Values of *p*<0.05 were considered statistically significant.

## RESULTS

The average age of the 165 subjects evaluated was 50 years with a SD of 10, the majority being female. There was a higher prevalence of patients with a partner, who belonged to a religion, had a high school education, and who were unemployed or removed by the INSS (Table 1).

**Table 1:** Sociodemographic characteristics of 165 patients with chronic pain at the Pain Clinic of C-HUPES/UFBA, from June 2016 To March 2019, Salvador-Bahia.

Variables	Results
Age in full years*	50 (10)
**Women**	148 (90%)
**Marital status** With companion	97 (59%)
No companion	68 (41%)
**Do you have a religion?**	157 (95%)
**Education** No schooling	2 (1%)
Complete/incomplete elementary school	64 (39%)
Complete/incomplete high school	81 (49%)
Complete/incomplete higher education	18 (11%)
**Labor activities** Employee	44 (27%)
Unemployed or removed by the INSS	104 (63%)
Retired	17 (10%)

Note:

* Values expressed as mean and standard deviation.

Most respondents reported moderate self-esteem and felt moderately satisfied with themselves. However, they had severe sleep disorders, severe pain, and the presence of stress with the resistance phase being the most prevalent. When asked to assess how they perceived the treatment, 58% revealed that they had improved (Table 2).

**Table 2:** Sleep quality, stress phases, and response to treatment of 165 patients related at the Pain Center of HUPES, from June 2016 to March 2019, Salvador-Bahia.

Variables	Results
**Sleep quality**	
Severe disorder	147 (89%)
Moderate disorder	4 (2%)
Mild disturbance	4 (2%)
Good sleep	10 (6%)
**Pain intensity (EVN)**	
Painless	2 (1%)
Light	4 (2%)
Moderate	54 (33%)
Intense	89 (54%)
Unbearable	16 (10%)
**Phases of stress**	
No stress	14 (9%)
Alert	10 (6%)
Resistance	83 (50%)
Near-exhaustion	46 (28%)
Exhaustion	12 (7%)
**Treatment evaluation**	
Improved	95 (58%)
Worsened	21 (13%)
Same thing	49 (30%)
**Self esteem**	
Bad/Bad	42 (26%)
Moderate	88 (53%)
Good/Great	35 (21%)
**Personal satisfaction**	
Nothing/Not very satisfied	42 (26%)
Moderately	77 (47%)
Well/Very	46 (27%)

Table 3 shows the patients distributed according to the different stages of stress. An association was observed between the phases of stress and self-esteem, depression, and anxiety showing that the greater the stress the worse the self-esteem and the more frequent the symptoms of depression and anxiety. Some associations could not be statistically analyzed due to the small number of individuals in some strata of the phases of stress (Table 3).

**Table 3:** Assessment of stress phases in 165 chronic pain patients treated at the Pain Clinic of C-HUPES/UFBA, from June 2016 to March 2019, Salvador-Bahia.

	Phases of Stress			
Variables	Almost Exhaustion/Exhaustion	Resistance	No Stress/Alert	*p*
	58 (35%)	83 (50%)	24 (15%)	
**Feminine gender**	49 (85%)	75 (90%)	24 (100%)	NA
**Marital status** No companion	>18 (31%)	37 (45%)	>13 (54%)	0,104
With companion	40 (69%)	46 (55%)	11 (46%)	
**Do you have a religion?**	55 (95%)	79 (95%)	21 (88%)	NA
**Education** No schooling	>0 (0%)	2 (2%)	>0 (0%)	
Elementary school	25 (43%)	32 (39%)	7 (29%)	NA
High school	27 (47%)	40 (48%)	14 (58%)	
University education	6 (10%)	9 (11%)	3 (13%)	
**Labor activities** Employee	>15 (26%)	25 (30%)	>4 (17%)	
Unemployed	37 (64%)	50 (60%)	17 (71%)	NA
Retired	6 (10%)	8 (10%)	3 (12%)	
**Treatment evaluation** Improved	>33 (57%)	48 (58%)	>14 (59%)	
Worsened	11 (19%)	8 (10%)	2 (8%)	NA
Same thing	14 (24%)	27 (32%)	8 (33%)	
**Self esteem** Bad/Bad	>24 (42%)	15 (18%)	>3 (12%)	
Moderate	28 (48%)	43 (52%)	17 (71%)	0,002*
Good/Great	6 (10%)	25 (30%)	4 (17%)	
**Personal satisfaction** **Nothing/Not very satisfied**	>20 (35%)	18 (22%)	>4 (17%)	
Moderately	28 (48%)	38 (46%)	11 (46%)	0,142
Well/Very	10 (17%)	27 (32%)	9 (37%)	
**Sleep quality** Severe Disorder	>55 (94%)	76 (92%)	>16 (67%)	
Moderate Disorder	1 (2%)	2 (2%)	1 (4%)	NA
Mild Disturbance	1 (2%)	2 (2%)	1 (4%)	
Good sleep	1 (2%)	3 (4%)	6 (25%)	
**Anxiety**	52 (90%)	61 (74%)	13 (54%)	0,002*
**Depression**	44 (76%)	46 (55%)	8 (33%)	0,001*

Notes: NA: Not applicable. The statistical test cannot be applied because it does not meet the assumptions;

* Pearson chi-square test.

## DISCUSSION

This study revealed that almost all patients with chronic pain had severe sleep disorders and stress with the resistance phase being the most prevalent. In addition, it was observed that the greater the stress, the worse the self-esteem and the more frequent the symptoms of depression and anxiety were.

The results found in this study are compatible with those in the literature regarding the higher prevalence of women with a partner, who have religion, have a high school education, and are away from work due to pain^15,16^. The significant prevalence of women found in this sample is related to the high frequency of gender, not only in this center, but also in studies on chronic pain in Brazil^17^. This is justified by the lower bone and muscle mass index and hormonal variations, in addition to the fact that in general women tend to carry out numerous work activities within and outside the home^18,19^.

Most subjects reported having difficulty or the inability to return to work. Other studies showed that most patients affected by pain have absenteeism, presenteeism, or are on sick leave due to illness^20^.

In this study, a high prevalence of severe and moderate sleep disorders was found, which confirms the findings in the literature that individuals with chronic pain have significant complaints regarding sleep quality^21^. Even with significant clinical efforts to assess and treat sleep disorders in patients at the center, it seemed very difficult to adequately control this variable when there is chronic pain.

Researchers have shown that not having restful sleep directly affects the individual’s worsening pain and increase in depression, anxiety, and stress^22^. Non-restorative sleep is also closely related to an increase in cortisol levels and, consequently, to higher levels of stress. Therefore, the decrease in hours slept directly reflects pain indexes. Sleeping less than the recommended number of hours can cause significant loss in health, as a whole, increasing in turn the painful perception^23^.

The fact that the assessed subjects report moderate self-esteem and personal satisfaction reflects the limitations that arise from chronic illness. The reduced functional capacity and the presence of anxious and depressive symptoms directly interfere with self-esteem and satisfaction within themselves^24^. However, treatment in a referral center, with a multidisciplinary team, may have been the support differential that facilitates a more positive response to these issues.

The findings in this study of most subjects are in the phase of resistance stress and exhaustion are compatible with some authors, who analyze that this may be due to the fact that pain and emotional suffering are closely related^25,26^.

Thus, more intense phases of the symptoms of stress seem to be related not only to the worsening of the pain, but also to the worse alteration in the sleep pattern and a greater presence of anxious and depressive symptoms. Studies show a close relationship between the increased response to pain and the worsening of depressive conditions and vice versa. This may be due to the fact that depression is a disease that causes the subject to harbor negative beliefs about himself, which manifests the low adherence to the proposed treatments, as well as the withdrawal from the social relationship^27-30^.

The withdrawal from social activities and increasing challenges in maintaining safe bonds with family members and spouses also stems from the limiting factors caused by chronic and persistent pain. Thus, the patient with pain keeps himself isolated in his family and social spaces, which contributes to the worsening of pain and mood in general^31^.

Thus, it is essential to understand how the subject with chronic pain deals with stress responses, the interference of these responses in the intensity of the pain, in the responses of the sleep cycle and in his mood, so that they can be better evaluated with a multidisciplinary team interdisciplinary that provides a careful approach, and favors a more effective social, professional, interpersonal relationship despite the pain^32^.

We believe that treating stress and controlling sleep can improve coping with pain. Studies that test the importance of the treatment of stress and sleep in people suffering from chronic pain would be interesting. We suggest that patients with chronic pain should be treated by a multidisciplinary team with different therapeutic measures to manage sleep stress and pain.

A limiting factor of the present study is the choice of the methodological design, which does not allow associations between the different outcomes, as it contemplates a specific photographic analysis. Another limitation is the heterogeneous nature of the sample regarding the etiology of pain. On the other hand, a positive factor is the fact that the study was carried out in a renowned reference center in our country, which minimizes diagnostic errors and favors a broad and interdisciplinary therapeutic approach.

Taking care of this patient, providing an active listening to the family and dialoguing with the health team means that the subject is evaluated and treated in an individualized way from his personal stories, losses obtained throughout life by pain and his suffering is recoded with new senses.

In conclusion, patients with chronic pain have impaired sleep quality a high level of stress and increased symptoms of anxiety and depression.

## Data Availability

Data sharing is not applicable to this article as no new data were created or analyzed in this study.
